# The course of physical functioning in the first two years after stroke depends on peoples’ individual movement behavior patterns

**DOI:** 10.1177/17474930211006293

**Published:** 2021-04-07

**Authors:** Roderick Wondergem, Martijn F Pisters, Eveline JM Wouters, Rob A de Bie, Cindy Veenhof, Johanna MA Visser-Meily

**Affiliations:** 1 Center for Physical Therapy Research and Innovation in Primary Care, Julius Health Care Centers, Utrecht, The Netherlands; 2 Department of Rehabilitation, Physical Therapy Science and Sport, Brain Center, University Medical Center Utrecht, University Utrecht, the Netherlands; 3 Research Group Empowering Healthy Behaviour, Department of Health Innovations and Technology, 3170 Fontys University of Applied Sciences, Eindhoven, the Netherlands; 4 Department of Health Innovations and Technology, 3170 Fontys University of Applied Sciences, Eindhoven, The Netherlands; 5 7899 Tilburg University, School of Social and Behavioral Sciences, Department of Tranzo, Tilburg, The Netherlands; 6 5211 Maastricht University, Department of Epidemiology and Caphri research school, Maastricht, The Netherlands; 7 Expertise Center Healthy Urban Living, Research Group Innovation of Human Movement Care, University of Applied Sciences Utrecht, Utrecht, the Netherlands; 8 Center of Excellence for Rehabilitation Medicine, Brain Center, University Medical Center Utrecht and De Hoogstraat Rehabilitation, Utrecht, The Netherlands

**Keywords:** Stroke, physical activity, sedentary behavior, physical functioning, movement behavior, secondary prevention

## Abstract

**Background and purpose:**

Deterioration of physical functioning after stroke in the long term is regarded as a major problem. Currently, the relationship between “peoples'” movement behavior patterns (the composition of sedentary behavior and physical activity during waking hours) directly after stroke and the development of physical functioning over time is unknown. Therefore, the objectives of this study were to investigate (1) the course of physical functioning within the first two years after returning home after stroke, and (2) the association between physical functioning and baseline movement behavior patterns.

**Method:**

In the longitudinal RISE cohort study, 200 persons with a first-ever stroke discharged to the home-setting were included. Participants’ physical functioning was assessed within three weeks, at six months, and one and two years after discharge using the Stroke Impact Scale (SIS) 3.0 subscale physical and the five-meter walk test (5MWT). Three distinct movement behavior patterns were identified in a previous study at baseline and were used in the current study: (1) *sedentary exercisers* (sufficiently active and 64% of waking hours sedentary)*,* (2) *sedentary movers’* (inactive and 63% of waking hours sedentary)*,* and (3) *sedentary prolongers* (inactive and >78% of waking hours sedentary accumulated in long prolonged bouts)*.* The association between movement behavior patterns and the course of physical functioning was determined using longitudinal generalized estimating equations analyses.

**Results:**

Overall participants’ physical functioning increased between discharge and six months and declined from six months up to two years. Physical functioning remained stable during the first two years after stroke in *sedentary exercisers*. Physical functioning improved during the first six months after discharge in *sedentary movers* and *sedentary prolongers* and deteriorated in the following six months. Only physical functioning (SIS) of *sedentary prolongers* further declined from one up to two years. A similar pattern was observed in the 5MWT.

**Conclusion:**

Movement behavior patterns identified directly after returning home in people with stroke are associated with and are predictive of the course of physical functioning. Highly sedentary and inactive people with stroke have unfavorable outcomes over time than individuals with higher amounts of physical activity.

## Background

Physical functioning after stroke is an important determinant for social reintegration.^1^ Deterioration of physical functioning is regarded as a major problem, as it could lead to dependency in daily life and participation restrictions.^2–4^ Over 50% of people with stroke report longer-term problems with physical functioning aspects, such as mobility and falls.^5^ Moreover, physical functioning declines over time after stroke in a substantial part of the population. Over 25% of all people with stroke decline in physical functioning within the first year after stroke,^2^ increasing to 40% percent in the first three years after the event.^6^ Therefore, prevention of deterioration of physical functioning in people with a first-ever stroke is important.

A sufficient amount of moderate to vigorous physical activity (MVPA) is associated with improved physical functioning after stroke^7,8^ and physical inactivity with decreased physical functioning.^9^ Although a sufficient amount of MVPA is protective for a decrease in physical functioning, it accounts for a small proportion of the day (<5%). Ignoring the other components of the movement continuum (sedentary behavior (SB) and light physical activity (LPA)) limits the understanding of how habitual movement behavior interacts to impact physical functioning. Therefore, “individuals'” movement behavior patterns, the composition of all levels of physical activity(light, moderate and vigorous) and SB during waking hours,^10^ and their relationship with the course of physical functioning over time need to be explored.

Our research group recently investigated the most commonly distinct movement behavior patterns in stroke patients, and three different groups of patients with distinct movement behavior patterns emerged: s*edentary exercisers (22%), sedentary movers (46%) and sedentary prolongers (32%).*^11^ Sedentary exercisers were sedentary for 64% of their waking hours and spent 27% of their waking hours in LPA and 10% in MVPA. During 63% of their waking hours, sedentary movers were sedentary, spent 34% in LPA and 3% in MVPA. Both sedentary exercisers and sedentary movers interrupted their SB frequently with physical activity. The third pattern, sedentary prolongers, were highly sedentary (78%), spent 20% of their time in LPA and 2% in MVPA. Sedentary prolongers spent their sedentary time in long prolonged bouts (≥30 min of uninterrupted sedentary behavior).

Based on previous literature in an older adult population, it could be expected that replacing sedentary behavior with LPA and MVPA will be associated with less loss of physical functioning.^12,13^ Currently, research investigating the course of physical functioning and the relationship with movement behavior patterns in people with stroke is lacking. Therefore, this study’s objectives were to investigate (1) the course of physical functioning within the first two years after returning home after stroke, and (2) the association between physical functioning and baseline movement behavior patterns.

## Methods

This is the prospective study based on the RISE longitudinal cohort study. The RISE study includes 200 persons with a first-ever stroke who were discharged from hospital to the home-setting.^11^ Participants from four stroke units in The Netherlands were included between February 2015 and April 2017. Their clinician asked eligible participants to participate if they had a clinically confirmed first-ever stroke and expected to return home with or without inpatient rehabilitation before returning home. Inclusion criteria were that they were independent regarding their activities of daily living (ADL) before stroke (Barthel index score >18^14^), over 18 years old, able to keep a conversation going (Utrecht Communication Assessment score >4^15^) and at least able to walk with supervision after stroke (Functional Ambulation Categories score >2^16^). People with subarachnoid hemorrhage were excluded. Written informed consent was obtained at the stroke unit. The study was approved by the Medical Ethics Research Committee of the University Medical Centre Utrecht (study number 14/76). After written informed consent was obtained, the demographic, stroke, and care characteristics were extracted from patients’ records. Participants were visited within three weeks, after six months, one year and two years after returning home. Physical functioning outcomes were obtained during the visits, and participants were asked if they received physiotherapy care. Movement behavior outcomes were obtained after the first visit by asking participants to wear an accelerometer for 14 days.

### Physical functioning

Physical functioning was measured with the subdomain, physical functioning, of the Stroke Impact Scale (SIS-physical) 3.0^17,18^ and the 5-m walking test (5MWT). Subdomains of the SIS 3.0 can be evaluated separately and show excellent validity.^19^ The subdomain physical functioning consists of 10 questions regarding ADL, eight regarding mobility, and five regarding hand function.^17,18^ As recommended, scores were calculated as percentages of the total amount of points, resulting in a range from 0 to 100. Lower scores indicate lower levels of physical functioning.

Walking speed was measured with the 5MWT^20^ because it was not possible to perform the 10-m walking test in some of the participants’ residences. Participants were asked to perform this test three times. The mean test time was calculated. The 5MWT has the same psychometric properties as the 10 MWT.^20^ A higher score on the 5MWT reflects a lower walking speed.

### Movement behavior

In the current study, participants are classified as one of three different movement behavior pattern groups observed within the first three weeks after returning home, as identified in earlier research by our group^11^: *sedentary exercisers*, *sedentary movers*, and *sedentary prolongers*. In this previous study, movement behavior patterns were identified by means of principal component analysis to compress 10 movement behavioral variables as recommended by Byrom et al. (e.g. mean time spent sedentary (hours/day)), LPA (hours/day), and MVPA (hours/day), mean time spent in sedentary bouts (uninterrupted periods of sitting and/or lying down) ≥5 minutes per day, ≥30 minutes per day and ≥60 minutes per day, meantime MVPA in bouts ≥10 minutes, weighted median sedentary bout length, maximum sedentary bout length and fragmentation index).^21^ The remaining components were used to identify movement behavior patterns using a k-means clustering algorithm, resulting in the three movement behavior patterns mentioned: *sedentary exercisers*, *sedentary movers* and *sedentary prolongers*.^11^

Movement behavior was measured using the Activ8 accelerometer, which was validated in community living ambulatory people with stroke.^22^ The Activ8 is a thigh-worn three-axial accelerometer. Participants received clear wearing instructions and registered wearing time on an activity log for fourteen days. The Activ8 measures 3D accelerations and derives postures and MET values from these data. The Activ8 data was provided in excel. The Activ8 measures with a frequency of 12.5 Hz, with a sample interval of five seconds, and stores a summary of the different postures and MET values every 5 min.^23^ In this study, five movement behavior outcomes were calculated at baseline: mean time of SB (sitting or lying position during waking hours <1.5 METs), LPA (1.5–3.0 METs), MVPA (≥3.0 METs),^10^ MVPA accumulated in bouts ≥10 min, and weighted median sedentary bout length. Mean time spent in SB, LPA and MVPA gives insight into the distribution of movement behavior during waking hours. MVPA accumulated in bouts ≥10 min is more beneficial for health.^24^ Therefore, the mean MVPA time accumulated in bouts ≥10 minutes was calculated as 10 or more consecutive MVPA minutes, with allowance for interruptions of no more than 2 min.^25^ To investigate prolonged SB, the weighted median sedentary bout length was calculated. The weighted median sedentary bout is the sedentary bout that corresponds with 50% of the total sedentary time.^21^

### Descriptive characteristics

Age, sex, BMI and presence of physiotherapy care were obtained from the medical record of each participant. Physiotherapy care after stroke was inventoried by asking the participant and/or relative at baseline and 6 months, one year and two years after discharge if they had received physiotherapy. Stroke severity was measured with the National Institutes of Health Stroke Scale (range 0–42) and was divided into: (1) no stroke symptoms (0 points), (2) minor stroke symptoms (1–4 points), and (3) moderate to severe stroke symptoms (≥5 points).^26,27^ Cognitive functioning was assessed with the Montreal Cognitive Assessment (range 0–30; <26 indicating impaired cognitive function).^28^ Balance was tested with the Berg Balance Scale (range 0–56, higher scores indicate better functioning).^25^ Activity limitations and participation restrictions were assessed using the Late-Life Function and Disability Instrument Computerized Adaptive Test (LLFDI-CAT) (scores range from 0 to 100, higher scores indicating better functioning or participation).^27^ The LLFDI-CAT consists of 137 questions for the activity limitations domain and 55 in the participation restriction domains. Questions are selected based on the answer to the preceding question. The stopping rule per domain was set for 10 questions.

### Statistical analyses

Normality assumption was checked by comparing histograms to a normal probability curve. Missing data were considered missing at random because data were more often missing for female participants. Therefore, multiple imputation using Multivariate Imputation by Chained Equation was used.^28^ Multiple imputation was performed by fitting models to predict missing physical functioning outcomes based on all other observed variables, including descriptive and movement behavior outcomes. Five imputed data sets were created and combined with a pooled set using “Rubins rules”.^29^

To study the course of physical functioning per movement behavior pattern, longitudinal analyses using generalized estimating equations (GEE) analyses were performed^30^ using an exchangeable correlation structure.^30^ Recovery patterns after stroke are known to reach a plateau in up to six months, and in the chronic phase, different trajectories occur (e.g. decline, remain stable, or increase).^9,31^ Therefore, different time periods were examined; from discharge to six months, from six months to one year, and from one year up to two years. For each outcome, a GEE was created to examine the course during each time period.

To study the association between movement behavior patterns and the course of physical functioning during the first two years after stroke, GEE analyses were performed,^11^ and subanalyses were performed from discharge to six months and from six months up to two years. Change scores were used in the GEE analysis to correct for baseline outcomes. Change scores of physical functioning (SIS-physical or 5MWT) outcomes were set as dependent variables, and movement behavior patterns served as independent variables. Stroke severity, age, sex and receiving physiotherapy care were added to all models to adjust for confounding effects since associations with the course physical functioning are expected. Sedentary exercisers were set as a reference to study the association of change in physical functioning compared to sedentary prolongers and sedentary movers compared to sedentary movers and sedentary prolongers. Results are expressed as regression coefficients (B) with 95% CIs. A negative score implies a decline in physical functioning compared to sedentary exercisers with B units per time period regarding SIS-physical and regarding the 5MWT.

*P*-values of <0.05 were considered statistically significant. Activ8 data was transferred from excel to SPSS. All analyses were carried out using SPSS (version 25.0; IBM corp.; Armonk NY).

## Results

**Figure 1.**
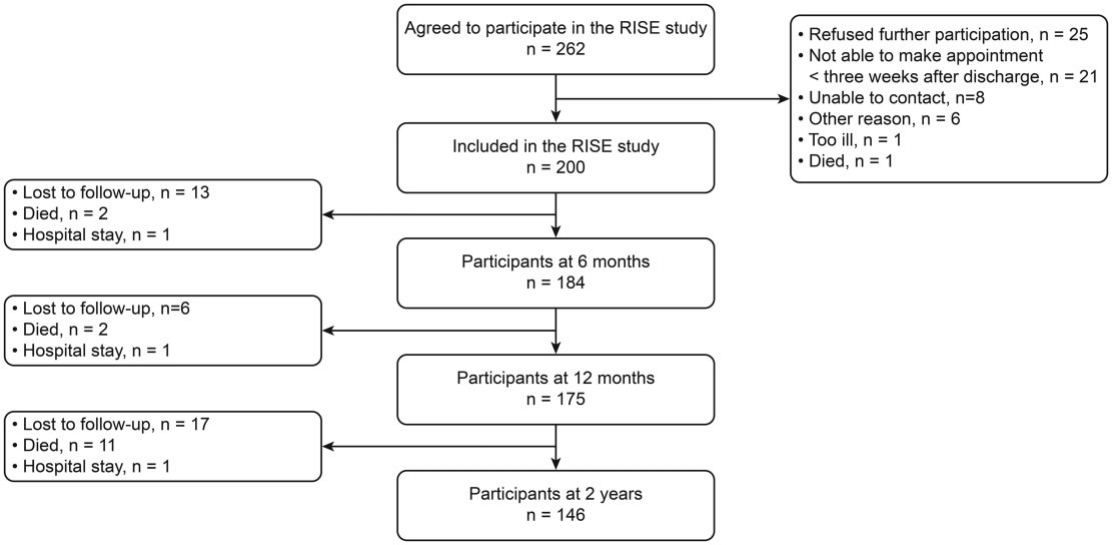
RISE – Study flow-chart.

A total of 262 people from the stroke-unit agreed to participate in the study. In total, 200 participants were included and analyzed. The flow-chart and reasons for refusal are presented in Figure 1. At six months, 184 (92%) people participated in the study, 175 (88%) after one year, and 146 (74%) after two years.

Table 1 presents baseline characteristics for the entire study sample after imputation of missing data. The mean age at the start of the study of the whole sample was 67.8 (SD 11.2) years. The majority of the population was male (64%), 68.5% had no or minor stroke symptoms, and 73.5% were discharged directly to the home-setting. Sedentary exercisers spent significantly more time in MVPA compared to the other two movement behavior patterns. Sedentary movers spent more time in LPA compared to the other two. Sedentary prolongers were more sedentary and spent less time in physical activity compared to the other two. Furthermore, they spent their sedentary time in long prolonged bouts. Differences between participants allocated to the different movement behavior patterns are shown in Table 1.

**Table 1.** Participant baseline characteristics and characteristics per movement behavior pattern expressed as the mean ± SD or percentage.

	Total group *n* = 200	Sedentary exercises *n* = 44 (22%)	Sedentary movers *n* = 90 (45%)	Sedentary prolongers *n* = 66 (33%)
Demographic factors				
Sex (male)	64.0	79.5	56.6^a^	63.6
Age (years)	67.8 ± 11.2	62.6 ± 11.2	69.3 ± 12.1^a^	69.3 ± 10.8^c^
Living together	76.3	72.7	72.2	83.3
Education level (high)	29.8	43.2	23.3	28.8
Stroke factors				
Ischemic stroke	91.5	93.2	90.0	92.4
Left hemisphere	53.5	56.8	48.8	57.6
Stroke severity (NIHSS)				
No stroke symptoms (0)	13.0	13.6	14.4	10.6
Minor stroke symptoms (1–4)	55.5	54.5	57.8	53.0
Moderate to severe stroke symptoms (>4)	31.5	31.8	27.7	36.4
Cognitively impaired (MOCA≤25)	59.0	61.4	58.9	57.6
Discharge destination				
Home	73.5	79.5	75.6	66.7
Rehabilitation	12.0	9.1	11.1	15.2
Geriatric rehabilitation	14.5	11.4	13.3	18.2
Physical functioning & participation				
Balance (BBS)	51.7 ± 6.6	55.1 ± 2.2	51.0 ± 6.7^a^	50.5 ± 7.6^c^
Activity limitations (LLFDI)	56.3 ± 11.6	64.6 ± 8.8	54.3 ± 11.6^a^	53.3 ± 10.6^c^
Participation restrictions (LLFDI)	46.8 ± 11.1	52.0 ± 9.1	45.8 ± 11.0^a^	44.7 ± 11.5^c^
Physical functioning (SIS)	83.9 ± 17.3	94.3 ± 6.8	82.9 ± 16.8^a^	78.4 ± 19.9^c^
Time in sec 5MWT	6.0 ± 3.7	4.5 ± 0.7	6.1 ± 3.3^a^	7.2 ± 4.8^c^
Movement behavior outcomes				
Sedentary time (hours)	9.3 ± 1.8	9.0 ± 1.6	8.4 ± 1.5	10.6 ± 1.4^b,c^
percentage	67.8 ± 10.9	63.6 ± 8.3	63.1 ± 10.5	77.1 ± 6.1^b,c^
LPA (hours)	3.8 ± 1.5	3.8 ± 1.2	4.6 ± 1.5^a^	2.8 ± 0.8^b,c^
percentage	27.6 ± 10.6	26.9 ± 7.9	33.6 ± 10.7^a^	20.0 ± 5.9^b,c^
MVPA (hours)	0.6 ± 0.5	1.3 ± 0.4	0.4 ± 0.3^a^	0.4 ± 0.3^c^
percentage	4.6 ± 4.1	9.5 ± 2.9	3.6 ± 3.9^a^	2.9 ± 2.1^c^
MVPA bouts ≥ 10 min	0.2 ± 0.3	0.7 ± 0.3	0.1 ± 0.1^a^	0.1 ± 0.1^c^
(hours)				
WMSB (minutes)	22.5 ± 13.5	15.8 ± 8.0	15.8 ± 7.3	36.1 ± 12.7^b,c^

Note: Values are percentage or mean ± SD.

NIHSS: National Institutes of Health Stroke Scale; MOCA: Montreal Cognitive Assessment; BBS: Berg Balance Scale; LLFDI: Late Life Function and Disability Index; SIS: Stroke impact scale; 5MWT: 5 meter walk test; LPA: light physical activity; MVPA: moderate-vigorous physical activity; WMSB: Weighted median sedentary bout length.

a Statistically significant differences between sedentary exercisers and sedentary movers.

b Statistically significant differences between sedentary movers and sedentary prolongers.

c Statistically significant differences between sedentary exercisers and sedentary prolongers.

### The course of physical functioning per movement behavior pattern

**Figure 2.**
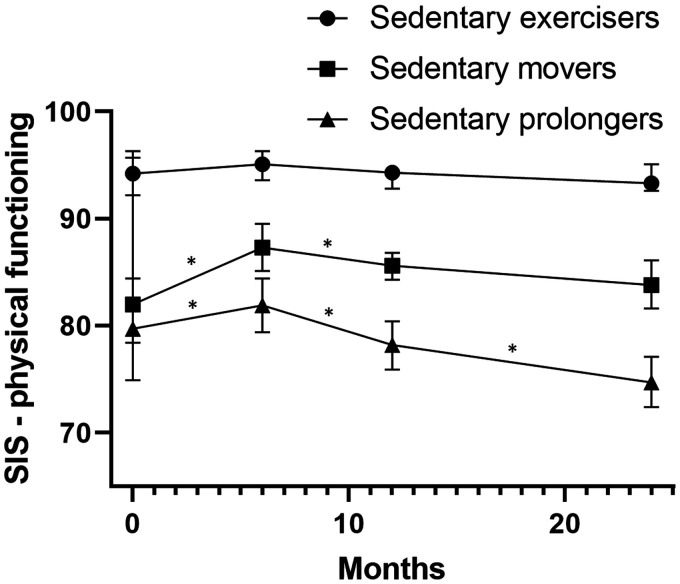
The course of physical functioning during the first two years after returning home in people with a first-ever stroke per movement behavior pattern objectified with the stroke impact scale 3.0 physical functioning. A lower score for the SIS physical functioning means a lower level of physical functioning. * Significant change between time points.

Table 2 presents the course of physical functioning in people with a first-ever stroke. Overall participants’ physical functioning increased between discharge and six months and decreased up to two years to a lower level compared to the level at six months (the highest level). Figures 2 (SIS-physical) and 3 (5MWT) present the course of physical functioning per movement behavior pattern. Sedentary exercisers’ physical functioning, measured with both the SIS-physical and the 5MWT, remained stable during the first two years after discharge.

**Table 2.** The course of physical functioning in the first year after discharge to home setting within the entire sample.

	Baseline mean (95% CI)	Mean change scores (95% CI) 6 months follow-up	Mean change scores (95% CI) 6 months to 1 year follow-up	Mean change scores (95% CI) 1 year to 2 years follow-up
SIS physical functioning	83.94 (81.55 to 86.34)	3.30 (1.97 to 4.63)^b^	−2.20 (−3.19 to −1.21)^b^	−2.13 (−3.46 to − 0.82)^b^
Time 5MWT (sec)^a^	6.01 (5.55 to 6.47)	−0.46 (−0.71 to −0.20)^b^	0.36 (0.07 to 0.65)^b^	0.42 (0.08 to 0.76)^b^

Note: Physical functioning outcomes are adjusted for stroke severity, age, sex and receiving physiotherapy care.

SIS: Stroke impact scale; ADL: Activities of daily living; 5MWT: 5-m walking test.

a A negative change means an improvement in the 5MWT time.

b Statistically significant change.

Sedentary exercisers’ baseline outcomes were significantly higher compared to both sedentary movers and sedentary prolongers. The baseline outcomes of sedentary movers’ and sedentary prolongers’ were not significantly different.

Sedentary movers’ physical functioning improved within the first six months after returning home and remained relatively stable for the long term. Only a small but significant decrease was observed between six months and one year after discharge measured with the SIS-physical.

Sedentary ‘prolongers’ physical functioning improved up to six months and declined between six months and one year and between one and two years. Regarding the 5MWT, the same pattern was observed, although the change was statistically significant only between one year and two years. Sedentary prolongers’ physical functioning declined to a lower level than the level at six months.

### The longitudinal association of physical functioning and the movement behavior patterns

Table 3 presents the association between movement behavior patterns and the course of physical functioning during the first two years after discharge to the home-setting and the subanalyses. In general, the analyses show that the course of physical functioning differs over time based on the movement behavior pattern at baseline.

**Figure 3.**
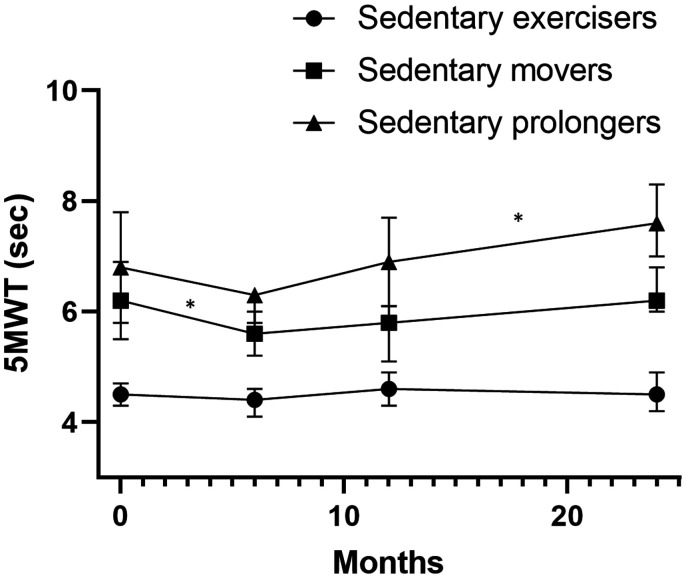
The course of physical functioning during the first two years after returning home in people with a first-ever stroke per movement behavior pattern objectified with the 5-m walk test. A higher score for the 5-m walking test reflects a lower walking speed. *Significant change between time points.

**Table 3.** The association between movement behavior patterns and change scores of physical functioning during the first two years after discharge, between discharge and first six months and between six months and two years after stroke using *sedentary exercisers* as a reference.

	Course between discharge and two years B (95%)	Course between discharge and six months B (95%)	Course between six months and two years B (95%)
SIS-Physical			
*Sedentary movers versus sedentary exercisers*	3.0 (0.8 to 5.1)^a,b^	2.0 (0.6 to 3.4)^a,b^	−0.1 (−1.6 to 1.3)
*Sedentary prolongers versus sedentary exercisers*	−0.7 (−3.2 to 1.7)	0.4 (−1.1 to 1.8)	−2.0 (−3.9 to 0.1)^a,b^
Walking speed (5MWT)			
*Sedentary movers versus sedentary exercisers*	−0.3 (−0.7 to −0.0)^a^	−0.3 (−0.5 to −0.1)^a^	0.1 (−0.2 to 0.4)
*Sedentary prolongers versus sedentary exercisers*	0.1 (−0.3 to 0.4)	−0.2 (−0.5 to 0.1)	0.4 (−0.3 to 1.1)

Note: Outcomes are adjusted for stroke severity, age, sex and receiving physiotherapy care.

B = Coefficient in GEE analysis (Interpretation: Difference on average over time in the course of physical functioning between movement behavior patterns. Compared to people with a “sedentary exercises” movement behavior pattern, physical function change over time is B units higher on average; negative signs (B) indicate a decline in physical functioning measured with the SIS-physical. A positive score for the 5 meter walking test means a decreased walking speed.)

B: unstandardized coefficient; CI: confidence interval; SIS: Stroke impact scale; 5MWT: 5 meter walking test.

a Difference with sedentary exercisers *P* < 0.05.

b Difference between sedentary movers and sedentary prolongers *P* < 0.05.

Sedentary movers improved their physical functioning more than both sedentary exercisers and sedentary prolongers up to two years measured with the SIS-physical. Subanalysis showed that the improvement took place between discharge and six months. Subanalysis showed that sedentary prolongers’ physical functioning declined more than sedentary movers and sedentary exercisers between six months and two years after discharge measured with the SIS physical. Although the same patterns were found when using the 5MWT as an outcome, only the improvement in walking speed of sedentary movers versus exercisers was statistically significant between discharge and two years and the subanalysis between discharge and six months.

## Discussion

The present study showed that physical functioning improves between discharge and six months and declines afterward up to two years. Physical functioning of the most active group, sedentary exercisers, remained fairly stable during the first year, while sedentary movers and sedentary prolongers improved during the first six months and declined afterward. Sedentary movers improved their physical functioning more within the first six months after discharge compared to sedentary exercisers and sedentary prolongers. Highly sedentary people have an unfavorable course of physical functioning over time compared to individuals with higher amounts of physical activity, including light-intensity activity.

Both baseline scores and the course of physical functioning differ between the movement behavior patterns. After discharge, sedentary prolongers had the lowest score, followed by sedentary movers and sedentary exercisers. Therefore, it seems that physical functioning outcomes at baseline are predictive for the course of physical functioning within the first two years after stroke. Sedentary exercisers’ physical functioning remained stable, while sedentary movers improved in the first six months and remained fairly stable onwards. In addition, prolongers improved, but less compared to movers, and declined more in the long term. Although the minimal clinical important differences (MCID) remove for SIS domain physical functioning are not yet determined, they have been for the subdomains ADL (5.9 points), mobility (4.5 points) and hand function (17.8 points). Therefore, the decline in physical functioning from six months up to two years in sedentary prolongers seems to be clinically important.^32^

The results underline the protective ability of sufficient amounts of MVPA, as sedentary exercisers are sufficiently active, and both sedentary movers and sedentary prolongers are inactive. MVPA is essential to improve and maintain physical fitness. Additionally, physical fitness determines our capacity to perform and tolerate physical activity and physical functioning.^33^ Because sedentary prolongers already had lower physical functioning outcomes at baseline and their course is worse than that of the other groups, the need for support to protect against a decline in physical functioning in this group is urgent. Recently, in a study with older adults, it was found that being less sedentary was related to less decline in physical functioning compared to older adults who spent more time in LPA.^12^ This is comparable to our results, as the sedentary movers had better outcomes. Although the amount of SB in sedentary movers is high, they spent more time in LPA than sedentary prolongers. Therefore, it seems that spending more time in LPA yields better physical functioning outcomes over time.

Movement behavior should therefore be investigated in all its aspects at the same time, in contrast to studies investigating only the amount of MVPA. When exclusively studying (sufficient) levels of MVPA, the benefits of increasing the level of LPA would have been underestimated. Our study’s results indicate that the course of physical functioning depends on peoples’ entire individual movement behavior in the first two years after discharge to the home-setting. More research will be needed to explore other factors that may have influence over the course of physical functioning and explore factors interacting with movement behavior.

Currently, the main focus in rehabilitation after stroke is on supervised training to improve fitness levels. However, it seems that movement behavior does not change over time.^34,35^ Therefore, sustainable behavioral change interventions to prevent decline in physical functioning are needed. Currently, interventions to improve unsupervised MVPA are poorly described, and intervention studies intended to address SB are scarce, while studies with a follow-up after three months are completely lacking.^36^ There is evidence that tailored counseling improves long-term physical activity participation, mainly when performed in the home setting of a person with stroke.^37^ Moreover, preliminary results of tailored interventions targeting SB reduction in older adults seem to be promising.^38^ Overall, research regarding the effectiveness of interventions targeting the reduction of SB in people with stroke is needed. In addition, thorough intervention descriptions in protocol articles are necessary to understand the effectiveness.

This study has several strengths. First, this study is the first longitudinal study investigating the association between baseline movement behavior and in physical functioning up to two years after returning home after first ever stroke.^10^ Second, although it could be questioned whether participants modified their movement behavior due to wearing an accelerometer, there are no studies known that have reported such effects using an accelerometer for 14 days. Therefore, we hold the opinion that the method used enables accurate assessment of habitual movement behavior of the participants at baseline.

Considering the limitations, the majority of the population (>90%) had an ischemic stroke, which is an overrepresentation of 15% compared to the stroke population in the Netherlands.^39^ This overrepresentation is probably caused by the fact that the majority of people with hemorrhagic strokes are referred to academic hospitals. Another limitation is that people with mainly minor stroke symptoms were included. However, as the baseline characteristics are comparable to another large sample in the Netherlands, we believe that the results are generalizable to a population of patients with stroke discharged to the home-setting.^40^ In this study movement behavior patterns were objectified in the first three weeks after returning home. Although movement behavior outcomes remain stable within the first two months after returning home in people with a first ever-stroke,^41^ individuals change movement behavior patterns. Therefore, future research should determine if individuals’ membership changes in movement behavior patterns within the first year after discharge to home.

In conclusion, movement behavior patterns, identified directly after returning home in people with a first-ever stroke, are associated with and are predictive of the course of physical functioning. Highly sedentary and inactive people with stroke have unfavorable outcomes over time than individuals with higher amounts of physical activity, including light-intensity physical activity. Improvements in daily habitual movement behavior might protect against deterioration of physical functioning. Therefore, tailored interventions, including behavioral movement change for *sedentary prolongers,* are needed.
